# Supplementary figures and images for: New Mechanistic Insights on Carbon Nanotubes’ Nanotoxicity Using Isolated Submitochondrial Particles, Molecular Docking, and Nano-QSTR Approaches
Source: Biology (Basel). 2021 Feb 25;10(3):171. doi: 10.3390/biology10030171 (PMC7996163; doi:10.3390/biology10030171)

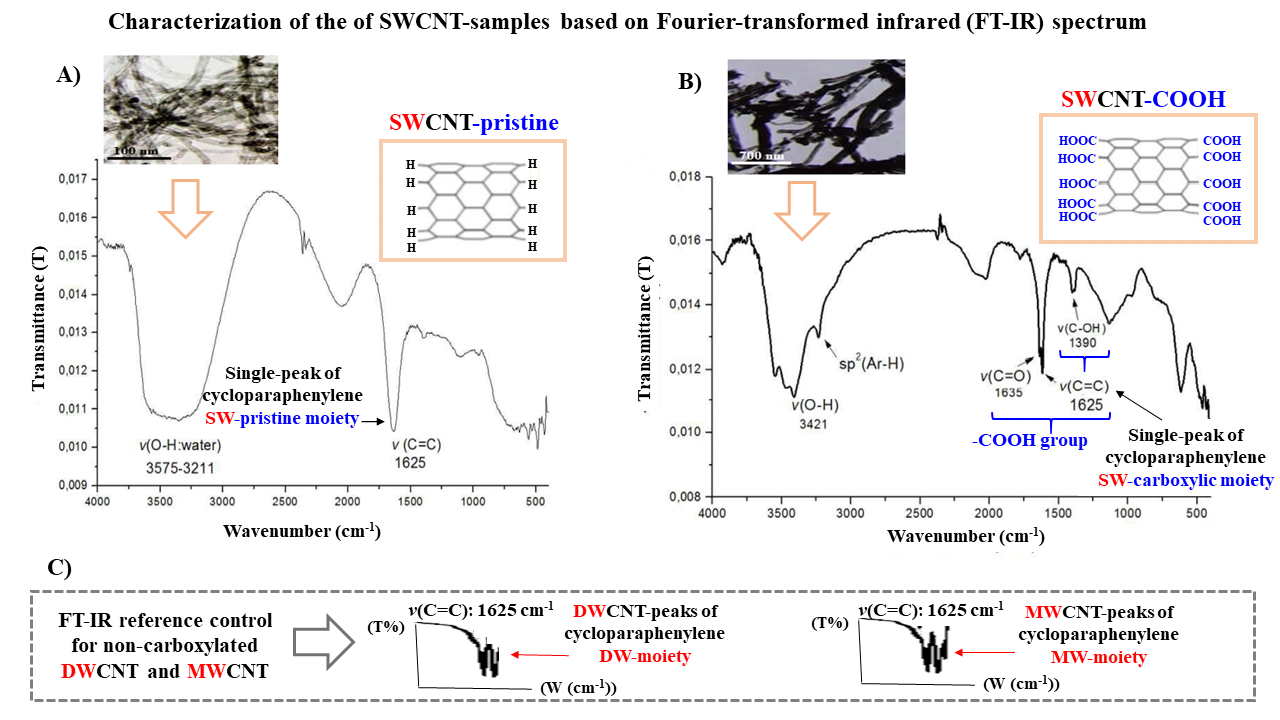

Supplement: Supplementary file 1 [file biology-10-00171-s001.tif]
